# Sweet Stress: Coping With Vascular Dysfunction in Diabetic Retinopathy

**DOI:** 10.3389/fphys.2018.00820

**Published:** 2018-07-13

**Authors:** Ana R. Santiago, Raquel Boia, Inês D. Aires, António F. Ambrósio, Rosa Fernandes

**Affiliations:** ^1^Coimbra Institute for Clinical and Biomedical Research, Faculty of Medicine, University of Coimbra, Coimbra, Portugal; ^2^CNC.IBILI, University of Coimbra, Coimbra, Portugal; ^3^Association for Innovation and Biomedical Research on Light and Image, Coimbra, Portugal

**Keywords:** diabetic retinopathy, blood–retinal barrier, retinal endothelial cells, oxidative stress, inflammation, apoptosis, neovascularization

## Abstract

Oxidative stress plays key roles in the pathogenesis of retinal diseases, such as diabetic retinopathy. Reactive oxygen species (ROS) are increased in the retina in diabetes and the antioxidant defense system is also compromised. Increased ROS stimulate the release of pro-inflammatory cytokines, promoting a chronic low-grade inflammation involving various signaling pathways. An excessive production of ROS can lead to retinal endothelial cell injury, increased microvascular permeability, and recruitment of inflammatory cells at the site of inflammation. Recent studies have started unraveling the complex crosstalk between retinal endothelial cells and neuroglial cells or leukocytes, via both cell-to-cell contact and secretion of cytokines. This crosstalk is essential for the maintenance of the integrity of retinal vascular structure. Under diabetic conditions, an aberrant interaction between endothelial cells and other resident cells of the retina or invading inflammatory cells takes place in the retina. Impairment in the secretion and flow of molecular signals between different cells can compromise the retinal vascular architecture and trigger angiogenesis. In this review, the synergistic contributions of redox-inflammatory processes for endothelial dysfunction in diabetic retinopathy will be examined, with particular attention paid to endothelial cell communication with other retinal cells.

## Introduction

Diabetic retinopathy is a leading cause of visual impairment in the working-age population of the Western world (Cheung et al., 2010). Of an estimated 468 million people with diabetes mellitus worldwide (Ogurtsova et al., 2017), more than 90 million diabetics have some form of diabetic retinopathy, of which one-third has severe vision-threatening diabetic retinopathy (Yau et al., 2012). Due to the increasing prevalence of diabetes, aging of the population, and increased life expectancy of those with diabetes, these numbers are expected to rise.

Diabetic retinopathy is very common among diabetic patients. Almost all patients with type 1 diabetes and more than 60% of patients with type 2 diabetes have some degree of retinopathy after 20 years with diabetes (Fong et al., 2004). In the Wisconsin Epidemiologic Study of Diabetic Retinopathy (WESDR), 3.6% of patients with type 1 diabetes and 1.6% of patients with type 2 diabetes were legally blind. In the group of patients with type 1 diabetes, 86% of blindness was attributable to diabetic retinopathy. In the group of type 2 diabetes patients, in which other eye diseases are common, diabetic retinopathy is responsible for one-third of the cases of legal blindness (Klein et al., 1984).

The classification of diabetic retinopathy into stages is based on the presence of visible ophthalmologic changes and the manifestation of retinal neovascularization. A first stage in diabetic retinopathy is preclinical retinopathy, without alterations in the eye fundus. Then, the disease progresses to mild non-proliferative retinopathy that is characterized by the presence of a few microaneurysms. The third stage is moderate non-proliferative retinopathy and it is characterized by the presence of microaneurysms, intraretinal hemorrhages, or venous beading. Microaneurysms and hemorrhages, identified as red dots, are the initial changes seen on ophthalmoscopic examination and fundus photography. The counting of red dots has been suggested as an appropriate marker of retinopathy progression (Klein et al., 1984, 1995). Microaneurysm formation is associated with localized proliferation of endothelial cells, loss of pericytes, and alterations of the capillary basement membrane (Cunha-Vaz, 1978). The fourth stage is severe non-proliferative diabetic retinopathy. In this stage, there is an increase in signs of retinal ischemia, which consists of cotton-wool spots, IRMA, several regions with lack of capillary perfusion, and significant venous beading. IRMAs are abnormal, dilated retinal capillaries that may represent forms of early neovascularization or forms of communication between arterioles and venules in zones of capillary occlusion. This stage is diagnosed using the “4-2-1 rule” of the Early Treatment of Diabetic Retinopathy Study, in which severe non-proliferative diabetic retinopathy is diagnosed if the patient has any of the following: diffuse intraretinal hemorrhages and microaneurysms in 4 quadrants, venous beading in ≥2 quadrants, or IRMA in ≥1 quadrant. Patients at this stage likely progress to the proliferative stage (Aiello, 2003). Proliferative diabetic retinopathy refers to a severe stage of diabetic retinopathy in which new blood vessels proliferate on the surface of the retina and posterior surface of the vitreous (Wu et al., 2013; Stitt et al., 2016). An important additional category in diabetic retinopathy is diabetic macular edema, which is an important manifestation of diabetic retinopathy and represents the most common cause of vision loss in patients with diabetes (Lee et al., 2015). Diabetic macular edema, characterized by retinal thickening resulting from leaky blood vessels, can develop at all stages of retinopathy, although it is more prevalent during the later phases (Antonetti et al., 2012).

The duration of diabetes is largely associated with the development and progression of retinopathy. The risk of diabetic retinopathy is also attributable to glycated hemoglobin and diabetes duration, as good glycemic control reduces the progression of retinopathy (Stratton et al., 2001; Chew et al., 2014). In addition, other factors may contribute to the risk for developing diabetic retinopathy and disease progression, such as dyslipidemia, high blood pressure, and chronic inflammation (Cardoso et al., 2017).

Several options are available to treat diabetic retinopathy in its later stages, such as laser photocoagulation, intravitreal injections of anti-VEGF or corticosteroids, and surgery. Laser treatment is highly effective in preventing visual loss and preserving vision in patients with advanced diabetic retinopathy and diabetic macular edema, who are at risk if not treated (Deschler et al., 2014). It should be noted that laser photocoagulation is an invasive procedure and vision is rarely improved or restored. Intravitreal therapies with anti-angiogenic agents, such as anti-VEGF, have improved the prognosis for diabetic macular edema (Schmidt-Erfurth et al., 2017) and anti-VEGF treatment is now the first-line therapy for diabetic macular edema involving the central macula (Thomas et al., 2013). The use of corticosteroids for treating patients with diabetic macular edema is suggested as a second choice, particularly for patients not responding to anti-VEGF therapy (Schmidt-Erfurth et al., 2017). Vitreous hemorrhage is a frequent complication of diabetic retinopathy, and vitreoretinal surgery is indicated for cases of non-clearing vitreous hemorrhage from proliferative diabetic retinopathy in patients who did not have previous laser photocoagulation (Berrocal et al., 2016). Pars plana vitrectomy, the surgical technique usually employed, has been suggested as a potential treatment option for diabetic macular edema patients with vitreoretinal traction, although the procedure remains controversial in cases without traction.

The pathogenesis of the disease is complex and several vascular, inflammatory, and neuronal mechanisms are known to be involved. The hallmark of diabetic retinopathy is the dysfunction of the inner BRB leading to increased permeability of serum constituents that may gain access to neural tissue. Genetic, metabolic, and environmental factors contribute to the four known biochemical pathophysiological pathways: polyol, AGEs, hexamine, and PKC. The alterations in these mechanisms can lead to hypoxia, oxidative stress, and inflammation that contribute to the breakdown of the BRB.

## Microvascular Alterations in Diabetic Retinopathy

**FIGURE 1:**
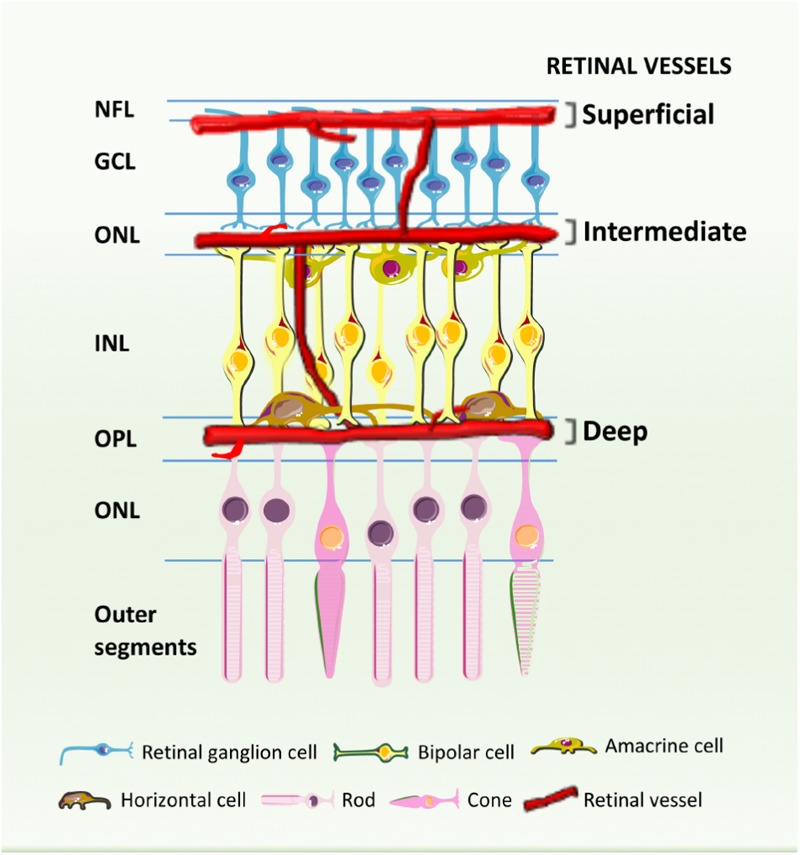
Schematic cross-section showing the retinal blood vessels lining the inner surface of the retina. Three capillary plexuses are embedded among retinal neurons: the superficial layer lies within the inner nerve fiber layer (NFL) with branches extending into the ganglion cell layer (GCL), while the intermediate and deep capillary plexuses align along each sides of the inner nuclear layer (INL). ONL, outer nuclear layer. IPL, inner nuclear layer; OPL, outer plexiform layer.

Historically, diabetic retinopathy has been thought to primarily affect the microvasculature (Lynch and Abramoff, 2017) probably due to the fact that the mainstay tests for screening for diabetic retinopathy predominantly detect microvascular changes. Besides the presence of microaneurysms and soft exudates, macular edema, and neovascularization (which are visible by ophthalmoscopy or fundus photography), it is now known that other retinal cell types are affected by diabetes and may compromise or contribute to visual impairment. In fact, diabetic retinopathy is characterized by progressive changes in the retinal microvasculature with accompanying neuroglial damage (Fletcher et al., 2007). The blood supply to the inner retina is composed of three different vascular plexuses embedded in the neural tissue: a layer of vessels that lies within the nerve fiber layer with branches extending into the ganglion cell layer, and two layers lying along each side of the inner nuclear layer (**Figure 1**). A recent study using OCTA, a non-invasive method of 3D imaging of the retinal and choroidal circulations, has revealed a more detailed vascular anatomy of human retina than previously described (Campbell et al., 2017). With a projection-resolved OCTA algorithm, an improved system of OCTA segmentation boundaries has been identified, with two complexes and four distinct vascular plexuses in the retina. The identification in the macula of an intermediate capillary plexus and deep capillary plexus at the level of deep vascular complexes may be useful in the assessment of vascular changes, such as capillary dropout, as well as abnormal vessels in all retinal layers in diabetic retinopathy (Campbell et al., 2017). This approach can provide earlier identification of capillary layers that are more affected in this and other retinal diseases, contributing to a deeper understanding of retinal pathophysiology.

The retinal vascular endothelium is a monolayer of cells covering the vascular lumen that supplies and drains the inner retina. On the one hand, the endothelium acts as a selective barrier (inner BRB) between the blood circulation and the neural retina. The presence of tight junctions prevents the outward flow of macromolecules and maintains the local microenvironment. On the other hand, the endothelium efficiently supplies oxygen and nutrients to the neural retina. However, under certain conditions, such as hyperglycemia, retinal endothelial cells become particularly vulnerable. Plasma glucose is transported across the cell membrane through facilitative glucose transporters, namely the constitutive isoform GLUT1 (Badr et al., 2000; Fernandes et al., 2003, 2004). At present, there is still conflicting data on GLUT1 protein regulation under high glucose conditions. Despite a localized increase in GLUT1 on the endothelial cells in more than half of the post-mortem retinas from patients with long-standing diabetes (>17 years) (Kumagai et al., 1996), no changes were observed in an animal model of long-standing diabetes (Fernandes et al., 2003), both using immunogold staining. However, a downregulation in total retinal GLUT1 and retinal microvascular GLUT1 was reported in alloxan- and streptozotocin-induced diabetic rats (Balabanov and Dore-Duffy, 1998; Badr et al., 2000; Fernandes et al., 2004). These contradictory results may be due to different species and duration of disease. Furthermore, in addition to GLUT1, studies on the contribution of other GLUT isoforms present in the BRB may provide a better understanding of retinal metabolism in diabetic retinopathy.

Most cells of the body when exposed to hyperglycemia are able to maintain their intracellular glucose levels relatively constant by regulating its transport into the cell. In contrast, endothelial cells struggle to control their intracellular basal glucose levels, and its increase can alter numerous cellular functions, leading to cell damage (Du et al., 2003; Kowluru and Abbas, 2003). Thus, diabetes damages endothelial cells and triggers a cascade of signaling events that leads to metabolic and biochemical derangements.

**FIGURE 2:**
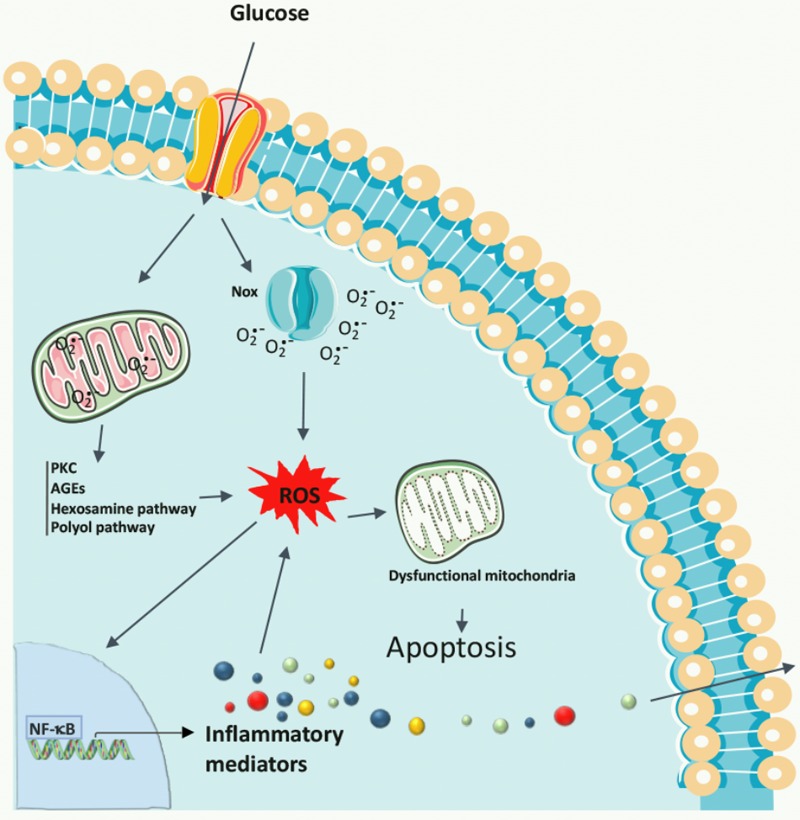
Biochemical pathways leading to diabetic retinopathy. Chronic hyperglycemia promotes the increase in ROS formation, originating in the mitochondria or produced by Nox, and also activates many metabolic pathways, including PKC, polyol pathway, AGEs, and the hexosamine pathway, as well as the production of inflammatory mediators. Oxidative stress damages mitochondrial DNA resulting in a decline in mitochondrial function, which accelerates cell apoptosis, contributing to the development of diabetic retinopathy.

A unifying mechanism of hyperglycemia-induced endothelial cellular injury was proposed by Brownlee (2005), in which the overproduction of superoxide anions by the mitochondrial electron transport chain during the process of oxidative phosphorylation is the common upstream event that may stimulate diverse biochemical pathways known to play a role in the pathogenesis of diabetic retinopathy: (i) increased flux through the polyol pathway and activation of aldose reductase (Lorenzi, 2007); (ii) enhanced non-enzymatic glycation; (iii) increased production of diacylglycerol; and (iv) stimulation of PKC (**Figure 2**). The overall effects of these mechanisms are an increase in ROS production and stimulation of the oxidative stress (Brownlee, 2005; Wu et al., 2014), resulting in a range of biochemical and metabolic abnormalities.

The overproduction of superoxide anion by the mitochondria under hyperglycemic conditions can leave an early imprint in cells of the vasculature, enabling the development of microvascular abnormalities even when good glycemic control is achieved. Superoxide anion has been suggested as a key player in this metabolic memory effect, and potential mechanisms for propagating this memory are the increased accumulation of potentially harmful agents, such as AGEs, which leads to glycation of mitochondrial proteins, lipids, and nucleic acids (Berezin, 2016). These injurious effects are not easily reversed after a reinstatement of good glucose control on hyperglycemia-induced oxidative stress, mainly if poor glycemic control is maintained for longer durations, and some alterations may be even permanent because of epigenetic changes (Ceriello, 2009). In fact, AGEs have been correlated with presence of varying stages of diabetic retinopathy, independent of HbA1c (Genuth et al., 2015). Furthermore, hyperglycemia-induced superoxide production, mainly from mitochondria, may lead to increased PKC activity and chronic low-grade inflammation mediated by activation of NF-κB. PKC activation leads to increased ROS generation via activation of mitochondrially localized adaptor protein Shc (p66^Shc^, which functions as a redox enzyme implicated in mitochondrial ROS generation and translation of oxidative signals into apoptosis) and NADPH oxidase signaling. Epigenetic changes, such as DNA methylation and acetylation and post-translational histone modifications, are responsible for persistent p66^Shc^ overexpression, promoting the metabolic memory and participating in diabetic retinopathy progression. Transient episodes of hyperglycemia-induced ROS formation in endothelial cells are sufficient to induce epigenetic changes responsible for perpetuating the metabolic memory by inducing sustained increase in the NF-kB subunit p65 gene expression and in the expression of p65-dependent pro-inflammatory genes (Perrone et al., 2014; Berezin, 2016). Despite the dysfunction of retinal mitochondria and impairment of the metabolic pathways (Sone et al., 1996), subclinical inflammation, dysregulation in cell–cell communication, and maintenance of cell homeostasis are also associated with early diabetes-related endothelial dysfunction.

Histological analyses of diabetic retinas have shown that loss of the retinal capillary pericytes (smooth muscle cells that provide tone to the vessels) and endothelial cells are two invariable features of the earliest events of diabetic retinopathy (Durham and Herman, 2011). Pericyte dropout, manifested by their degeneration (ghost cells), may eventually lead to the formation of acellular capillaries, microaneurysms with decreased retinal perfusion, capillary basement thickening, and induction of a series of biochemical and metabolic alterations (Barot et al., 2013). Non-vascular cells, such as Müller cells and other glial cells, also undergo apoptosis. Capillary occlusion and retinal ischemia cause increased expression and release of a number of growth factors and cytokines. These factors include VEGF, which increases vascular permeability and favors the growth of abnormal new vessels (Frank, 2004). In proliferative diabetic retinopathy, pathological angiogenesis driven by VEGF originating from pericytes, retinal ganglion cells, and glia has been implicated in irreversible vision loss (Frank, 2004). In fact, VEGF levels are significantly increased in both vitreous and aqueous fluids of patients with proliferative diabetic retinopathy compared with samples from patients with non-proliferative diabetic retinopathy or without diabetes (Aiello et al., 1994; Duh and Aiello, 1999). However, low levels of the pro-angiogenic factor VEGF in the presence of increased levels of angiopoietin-2 were associated with activation of apoptotic pathways, detrimental to survival and proliferation pathways, leading to endothelial cell death and vessel regression in the early stages of diabetic retinopathy (Bento et al., 2010). VEGF is likely to be part of the angiogenic paradox of diabetes, in which both pro- and anti-angiogenic conditions may coexist with chronic hyperglycemia, and in particular, in the time course of retinopathy.

Other agents are also involved in the development of pathological angiogenesis and include angiopoietin-1 and -2 (Joussen et al., 2002b; Hammes et al., 2004; Cai et al., 2008), erythropoietin (Chen et al., 2009; Hernandez and Simo, 2012), the renin–angiotensin–aldosterone system (Wilkinson-Berka et al., 2007), fibroblast growth factor 2, TNF, and stromal-derived factor 1 alpha and its receptor, CXCR4. Many of these factors act in concert to mediate the key steps leading to angiogenesis, including protease production, endothelial cell proliferation, migration, and tube formation (Grant et al., 2004). Furthermore, the balance between angiogenic and anti-angiogenic factors is a key factor in determining the development and progression of proliferative diabetic retinopathy (Simo et al., 2006; Lima e Silva et al., 2007).

## Reactive Oxygen Species and Oxidative Stress in Retinal Endothelial Cells During Diabetes

Glucose oxidation, high oxygen demand, and the presence of a high content in polyunsaturated fatty acids render the retina particularly susceptible to oxidative stress (Anderson et al., 1984). Under physiological conditions, ROS are necessary to support a variety of cellular processes, such as metabolism, proliferation, differentiation, immune system regulation, and vascular remodeling (Wilkinson-Berka et al., 2013). However, disturbances in the balance between the generation and rapid scavenging of ROS generate toxic effects that can result in cell death (Calabrese et al., 2007). Superoxide anion (O_2_^∙-^) and the hydroxyl radical (∙OH) are considered the primary ROS, since they are unstable and react with biological molecules, such as proteins, lipids, carbohydrates, and nucleic acids.

Increased oxidative stress is detected in the retinas of experimental animal models with diabetes and galactosemia (Barot et al., 2011; Fernandes et al., 2014). The primary sources of intracellular ROS are the mitochondrial electron transport chain, cytochrome P450, xanthine oxidase, NOS, and Nox (Bedard et al., 2007; Millar et al., 2007; Forstermann, 2008; Sorce et al., 2012; Takac et al., 2012; Wilkinson-Berka et al., 2013). Elevated levels of ROS can be found as a result of the compromised activity of ROS-detoxifying systems, such as reduced glutathione, vitamin E, superoxide dismutase, catalase, thioredoxin peroxidase, and heme oxygenase (Wilkinson-Berka et al., 2013).

In retinal endothelial cells, high glucose levels can lead to overproduction of mitochondrial ROS, which then activate the poly-ADP-ribose polymerase (PARP) pathway decreasing the activity of glyceraldehyde 3-phosphate dehydrogenase and the levels of sirtuin 1 (SIRT1, a class III histone deacetylase) (Zheng et al., 2012). Interestingly, SIRT1 downregulation that leads to sustained responses of inflammatory and apoptotic proteins, such as NF-κB, Bax, and PARP, was maintained and propagated in cultured retinal endothelial cells, even after 2 weeks of normal glucose following 1 week of exposure to high glucose concentrations (Zheng et al., 2012). A potential mechanism for propagating cellular metabolic memory linked to high glucose exposure involves an excess of ROS production from mitochondria, and SIRT1 has been identified as a key player in this memory of retinal endothelial cells (Ihnat et al., 2007; Zheng et al., 2012).

A growing body of evidence indicates that Nox enzymes play an important role in the development of diabetic retinopathy (Li et al., 2010; Rojas et al., 2013; Kowluru et al., 2014; Wang et al., 2014). The superoxide-generating Nox enzymes are a primary source of extra-mitochondrial ROS generation (Jiang et al., 2011; Rinnerthaler et al., 2012). Besides their major role in the production of ROS (mainly superoxide anion and hydrogen peroxide), Nox isoforms have pro-inflammatory effects in the retina. Nox1, Nox2, and Nox4 are three of seven isoforms (Nox1 to -5, Duox1, and Duox2) that are expressed in retinal endothelial cells (Li et al., 2010) and all these isoforms can stimulate endothelial cell proliferation and tubulogenesis. *In vitro* studies with retinal endothelial cells under hypoxic and high glucose conditions revealed an upregulation of mRNA expression and protein levels of Nox4, ROS generation, and VEGF levels. Inhibition of Nox4 activity by statins (lovostatin) downregulates hypoxia-inducible factor 1-alpha and STAT3-mediated VEGF expression and ameliorate retinal vascular leakage in diabetic retinopathy (Li et al., 2010). GKT137831 (member of the pyrazolopyridine dione family), a dual inhibitor of Nox1 and Nox4, reduced the increased gene and protein expression of VEGF, monocyte chemoattractant protein-1, and leukocyte adhesion molecules as well as vascular leakage in an experimental model of ischemic retina (Deliyanti and Wilkinson-Berka, 2015). These findings imply an important role of Nox1/4 in endothelial function via regulation of migration and infiltration of monocytes/macrophages and BRB breakdown. Among the three isoforms, Nox2 has been the widest studied since its role in phagocytic defense and inflammation in diabetic retinopathy has been well established. In fact, increased levels of Nox2 in retinal blood vessels were associated with increased oxidative stress in the retina in an experimental model of diabetic retinopathy. Deletion of Nox2 or apocynin (a selective Nox inhibitor) treatment prevented diabetes-induced increases in ROS and ICAM-1 levels as well as retinal leukostasis and vascular leakage, suggesting that Nox2 is a key player in pathological conditions characterized by retinal vascular inflammatory reactions (Al-Shabrawey et al., 2008). Additionally, hyperglycemia-induced endothelial damage can generate reactive nitrogen species, such as peroxynitrite (ONOO^-^), through the rapid reaction of superoxide anion with nitric oxide. Peroxynitrite is a highly potent oxidant and nitrosylating agent that promotes leukocyte adhesion to retinal vessels and induces BRB breakdown (Leal et al., 2007; Pacher et al., 2007; Goncalves et al., 2012).

## Inflammation in Diabetic Retinopathy

Diabetic retinopathy has been recognized as chronic inflammatory disease, and local inflammation has been indicated as a novel risk factor for its development and progression (Lee et al., 2015; Atchison and Barkmeier, 2016). The origin of the inflammatory environment in the retina during diabetes still needs clarification. Nevertheless, since retinal apoptotic cell death occurs in diabetic conditions that may trigger an inflammatory condition, some authors have proposed that the metabolic alterations are at the genesis of inflammation (Tang and Kern, 2011).

Inflammatory cytokines have a role in the pathophysiology of this disease. Inflammatory cytokines, such TNF, IL-6, and C-reactive protein, mainly produced by adipose tissue and macrophages, have been detected in the serum of type 2 diabetic patients (Ellulu et al., 2017) and were associated with the microvascular complications of diabetic retinopathy (Schram et al., 2005). However, local inflammation seems to be more relevant for the development of diabetic retinopathy. Several cytokines, chemokines, and other factors are increased in the retina and vitreous of diabetic patients and animal models of diabetes (Hernandez et al., 2005; Tang and Kern, 2011; Abcouwer, 2013).

Inflammation mediates molecular and structural alterations associated with diabetic retinopathy, such as the breakdown of the BRB. Inflammation is the basis for the treatment with corticosteroids. Glucocorticoids decrease the inflammatory processes and improve BRB function by inhibiting leukocyte recruitment (Tamura et al., 2005). Inflammation also plays a role in the development of diabetic macular edema due to the accumulation of leukocytes on the surface of capillaries, an early event in BRB breakdown (Leal et al., 2007). Interestingly, vitreous VEGF levels are not increased in all patients with proliferative diabetic retinopathy (Aiello et al., 1994), which may be the reason why some patients do not respond to anti-VEGF therapies (Singer et al., 2016). It has been suggested that in such non-VEGF responders, intravitreal steroid injection would be more appropriate because pro-inflammatory cytokines probably play a major pathological role (Corcostegui et al., 2017).

## Linking Inflammation and Oxidative Stress in Diabetic Retinopathy

Chronic hyperglycemia-associated oxidative stress and low-grade inflammation are considered to play critical roles in the onset and progression of the complications of diabetes, including diabetic retinopathy (Xu et al., 2009; Frey and Antonetti, 2011). The mechanisms by which oxidative stress triggers inflammation and inflammation induces oxidative stress have not been totally clarified. Oxidative stress increases the production of cytokines through the activation of the transcription factors, such as NF-κB, STAT, and activator protein-1 that lead to the transcription of genes encoding cytokines (Turpaev, 2002; Wilkinson-Berka et al., 2013). The activity of NF-κB has been demonstrated to be increased in endothelial cells, pericytes, and glial cells in experimental models of diabetic retinopathy, and in the retinas of diabetic patients (Romeo et al., 2002; Kowluru et al., 2003; Harada et al., 2006). The inhibition of NF-κB inhibits diabetes-induced production of inflammatory molecules in retinal tissue, highlighting the essential role of NF-κB in diabetes-induced retinal inflammation (Nagai et al., 2007).

Cytokines are also able to increase the production of ROS. TNF, IL-1β, and interferon-γ induce ROS production in retinal pigment epithelial cells. TNF and IL-1β increase ROS production via mitochondria and Nox, respectively. Interferon-γ elicits ROS production via mitochondria and Nox (Yang et al., 2007). The production of chemokine (C-C motif) ligand 2 during retinal vascular inflammation requires activation of the Nox pathway through NF-κB and Akt signaling pathways (Zhang W. et al., 2009). A convincing report further confirmed that cytokines trigger ROS production in the retina by demonstrating that IL-1β administration to the vitreous of experimental animals increases oxidative stress in the retina, similar to studies done in diabetes models (Kowluru and Odenbach, 2004a,b). Pro-inflammatory cytokines induce the formation of ROS and NO, which, through the formation of peroxynitrite, reduce the content of small heat shock protein 27 and lead to apoptotic cell death of retinal endothelial cells (Nahomi et al., 2014).

## Crosstalk Between Endothelial Cells and Circulating Mediators and Inflammatory Cells

Endothelial cells together with the regulatory pericyte and glial cells surrounding the retinal vessels are essential for maintaining BRB integrity and normal function. In diabetic patients and experimentally induced diabetic animals, death of pericytes and endothelial cells has been reported, and precedes histological evidence of retinopathy (Mizutani et al., 1996). Altered permeability of retinal capillaries that leads to the breakdown of the inner BRB is considered a hallmark of diabetic retinopathy (Klaassen et al., 2013). However, the molecular mechanisms underlying altered permeability of retinal endothelial cells remain to be clarified. Mounting evidence suggests that inflammation plays an important role in the development of diabetic retinopathy (Tang and Kern, 2011). In fact, elevated levels of pro-inflammatory cytokines have been detected in the vitreous of diabetic patients with retinopathy (Abu el Asrar et al., 1992) and in diabetic rat retinas with increased vascular permeability (Carmo et al., 2000; Joussen et al., 2002a).

It was demonstrated that TNF has an important role in the pathogenesis of diabetic retinopathy, being an important inducer of endothelial cell apoptosis (Robaye et al., 1991; Polunovsky et al., 1994). TNF also increases retinal endothelial cell monolayer permeability by inducing a decrease in the tight junction proteins zonula occludens (ZO)-1 and claudin-5 and a localization shift of both proteins (Aveleira et al., 2010). However, an apparent contradiction arose regarding if the presence of TNF is more important for BRB breakdown in the initial or late phases of diabetic retinopathy (Aveleira et al., 2010; Huang et al., 2011). Using a TNF-knockout mouse, it was observed that the absence of TNF had no effect in the initial phases of diabetic retinopathy-associated BRB breakdown, even though it prevented retinal leukostasis. At later phases, BRB breakdown was suppressed by the absence of TNF (Huang et al., 2011). Moreover, it was demonstrated that high glucose-induced cell death is due to the activation of TNF receptor 1 by TNF. Thus, blocking the activity of this receptor could be an adequate strategy to avoid cell loss in diabetic conditions (Costa et al., 2012). Additionally, TNF could be a good therapeutic target for the prevention of diabetic retinopathy progression (Zhang et al., 2011). Furthermore, it has been suggested that its concentration in the tear fluid could be used as a biomarker for the detection of diabetic retinopathy (Costagliola et al., 2013).

The cytokine IL-1β is increased in the vitreous of patients with diabetic retinopathy and has been implicated in disease progression (Mao and Yan, 2014). It was suggested that IL-1β contributes to accelerated apoptosis of retinal endothelial cells under hyperglycemic conditions (Kowluru and Odenbach, 2004b). In fact, hyperglycemia triggers the increase in IL-1β production in diabetic retinopathy that is mainly produced by retinal vascular endothelial cells (Liu et al., 2012). IL-1β itself induces an autostimulation in endothelial cells, which is the main mechanism for sustained IL-1β overexpression (Liu et al., 2012). It was pointed out that interrupting the IL-1β-induced autostimulation could limit the progression of diabetic retinopathy. IL-1β-induced autostimulation occurs via forkhead transcription factor (FoxO1) upregulation in endothelial cells, and silencing FoxO1 reduces the IL-1β expression levels (Wu et al., 2017).

When inflammation persists chronically, leukocytes that are recruited to the inflamed tissue can induce tissue damage by prolonged secretion of chemical mediators and toxic ROS (Noda et al., 2012), further contributing to the progression of diabetic retinopathy. In fact, during diabetes, leukocytes have been shown to be activated (Miyamoto et al., 1998; Kinukawa et al., 1999; Nonaka et al., 2000) and to become a source of Nox-derived ROS (Mohanty et al., 2000; Wong et al., 2002). Leukocytes, therefore, play a critical role in capillary degeneration and permeability and contribute to retinal edema, ischemia, and angiogenesis (Ishida et al., 2004). It was observed that leukocytes colocalize with dying endothelial cells in the diabetic retina (Schroder et al., 1991). Moreover, leukocytes from diabetic mice trigger the death of more retinal endothelial cells than leukocytes from non-diabetic mice (Li et al., 2012), and neutrophils from diabetic animals exhibit higher levels of surface integrin expression and integrin-mediated adhesion (Barouch et al., 2000). Also, leukocytes from diabetic patients were found to induce the death of human retinal endothelial cells, by a mechanism that is dependent on direct contact and indicates that cell surface molecules are involved (Tian et al., 2013). Blocking integrin ligand integrin beta-2 (CD18) on leukocytes or ICAM-1 on the surface of retinal endothelial cells inhibits the leukocyte-mediated death of endothelial cells (Barouch et al., 2000; Tian et al., 2013). Thus, therapies that block inflammation, as well as leukocyte–endothelial cell–cell interactions can be envisaged as potential treatments for diabetic retinopathy. In fact, in diabetic CD18- and ICAM-1-deficient mice there are fewer adherent leukocytes in the retinal vasculature, which is associated with fewer damaged endothelial cells and reduced vascular leakage (Joussen et al., 2004). The administration of berberine, which has anti-inflammatory and antioxidant properties, inhibits leukocyte adhesion to retinal endothelial cells, as well as the leukocyte-mediated death of endothelial cells by the downregulation of ICAM-1 and CD18 (Tian et al., 2013).

Calcium dobesilate has been prescribed for the treatment of diabetic retinopathy (Garay et al., 2005), reducing the progression of the disease (Zhang et al., 2015). In an animal model of diabetes, we reported that calcium dobesilate prevents the BRB breakdown induced by diabetes by preventing the alterations in the tight junction proteins and decreasing leukocyte adhesion to retinal vessels (Leal et al., 2010). The beneficial properties of calcium dobesilate partly rely on the decrease of pro-inflammatory processes and oxidative stress in the retina (Voabil et al., 2017). Sitagliptin, an oral anti-hyperglycemic drug of the dipeptidyl peptidase-4 inhibitor class (Drucker and Nauck, 2006), also prevents the breakdown of BRB, inflammation, apoptosis, and alterations in the tight junction complexes in an animal model of diabetes (Goncalves et al., 2014).

## Crosstalk Between Endothelial and Retinal Cells

### Pericytes

**FIGURE 3:**
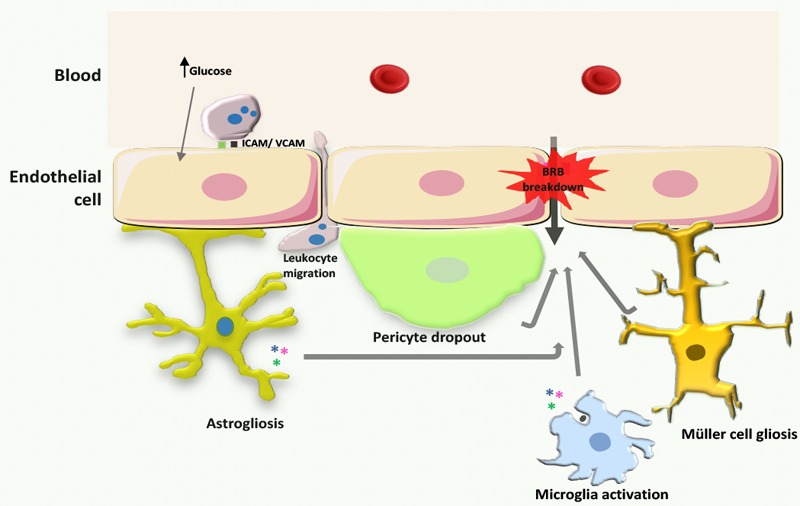
Processes leading to BRB breakdown in diabetic retinopathy. During the development of diabetic retinopathy, increased production of ROS leads to increased expression of intercellular adhesion molecules in endothelial cells, and consequently increased leukocyte adhesion. Concurrently, astrocytes, Müller cells, and microglia become activated, promoting cytokine release, and pericytes surrounding endothelial cells die by apoptosis. Moreover, vascular ROS and inflammatory mediators that are released by inflammatory cells affect endothelial tight junctions leading to increased BRB permeability.

The loss of pericytes is an early histopathological hallmark of diabetic retinopathy (Curtis et al., 2009). These cells have an important role in the maintenance of microvascular homeostasis, since they are involved in the maintenance of capillary tone and in the regulation of the vessel growth. In addition, pericytes also preserve the prostacyclin-producing ability and protect against lipid-peroxide-induced injury of the endothelial cells (Bergers and Song, 2005; Hamilton et al., 2010). Co-culture experiments have suggested that pericytes inhibit endothelial cell proliferation in a contact- or proximity-dependent manner (Orlidge and D’Amore, 1987). Also, pericytes increase the barrier properties of endothelial cell monolayers (Balabanov and Dore-Duffy, 1998), through transforming growth factor-beta (Dohgu et al., 2005) production. Pericytes provide paracrine signals that promote vessel stabilization. A pericyte-derived angiopoietin-1 multimeric complex binds to the Tie2 receptor on endothelial cells, inducing the expression of the tight junction protein occludin (Hori et al., 2004) *in vitro*. Therefore, the loss of pericytes could predispose the vessels to angiogenesis, thrombogenesis, and endothelial cell injury, leading to the clinical manifestations of diabetic retinopathy. Retinal pericytes accumulate AGEs during diabetes, altering their function and decreasing cell survival with harmful consequences to the BRB, leading to the progression of diabetic retinopathy (**Figure 3**). Indeed, the interaction between AGEs and their receptors (known as RAGEs) elicits ROS generation in cultured retinal pericytes, thereby inducing apoptotic cell death (Yamagishi et al., 2005). AGEs induce NF-κB activation, decrease the ratio of Bcl-2/Bax, and subsequently increase the activity of caspase-3, a key enzyme in the apoptosis of pericytes. Moreover, AGEs upregulate RAGEs expression levels in pericytes via ROS production (Yamagishi, 2011). These positive feedback loops transduce the AGE signals, thus further exacerbating their cytotoxic effect. Interestingly, it has been suggested that the loss of pericytes does not directly influence BRB barrier properties but, on the other hand, sensitizes endothelial cells to the effects of VEGF-A and angiopoietin-2, thus leading to changes in vascular permeability and angiogenesis (Park et al., 2014, 2017). In the absence of pericytes, the endothelial cells lose their regulation of proliferation and form new vessels (Beltramo and Porta, 2013). Therefore, the crosstalk between pericytes and endothelial cells is essential for vascular function and retinal homeostasis.

### Astrocytes

Astrocytes are glial cells present in the retina enveloping ganglion cell axons and forming axonal and vascular glial sheaths. These cells are active part of the BRB, sensing alterations in neuronal function and controlling the transfer of components from blood vessels to neurons. In fact, astrocytes modulate retinal homeostasis by nourishing neurons with glucose and by regulating extracellular potassium levels and metabolism of neurotransmitters. Although less abundant in retinal tissue than Müller cells, astrocytes play a crucial role in the transmission of retinal signals by retinal ganglion cells to the superior colliculus in the brain. In response to noxious stimuli, astrocytes suffer astrogliosis, a process characterized by changes in morphology, an increase in their proliferation rate, and the expression of glial fibrillary acidic protein (de Hoz et al., 2016) (**Figure 3**). In high glucose conditions, astrocytes proliferate and change their motility and distribution due to an increase in the expression of adhesion molecules (Shin et al., 2014). In addition, in diabetic retinopathy, astrocytes increase the expression of NF-κB (Shin et al., 2014) and increase the inflammatory milieu with direct consequences on the properties of the BRB (Barber et al., 2000). Also, the increase in angiopoietin-2 produced by endothelial cells in the diabetic retina (Yao et al., 2007) induces astrocyte apoptosis and BRB impairment (Yun et al., 2016).

### Müller Cells

Müller cells are the most abundant glial cells in the retina and constitute an anatomic and functional link between neurons and blood vessels, spreading from the subretinal space to the vitreous body. Therefore, Müller cells play a very important role in the nourishment of retinal neurons through their direct link with blood vessels. In addition, by sensing alterations in neural activity, Müller cells are able to buffer the levels of neurotransmitters such as glutamate and ions like potassium and also dispose of metabolic waste derived from the activity of photoreceptors and other retinal neurons. Furthermore, Müller cells regulate vascular tone and have a structural function in the integrity of the BRB (Tout et al., 1993). Alterations in retinal homeostasis lead to Müller cell gliosis, a process characterized by a shift in the supportive role of Müller cells toward a pro-inflammatory state with loss of function. In the diabetic retina, there is an imbalance in glutamate homeostasis (Santiago et al., 2006, 2008, 2009), which may be due to decreased uptake by Müller cells caused by an impairment in glutamate-aspartate transporter and glutamine synthetase activity. The decreased function of glutamate-aspartate transporter and glutamine synthetase lead to an extracellular accumulation of glutamate and lack of glutamine needed for the synthesis of gamma-Aminobutyric acid and glutamate. The accumulation of extracellular glutamate induces neurotoxicity and oxidative stress in retinal neurons culminating in irreversible neural cell loss. Besides their role in glutamate excitotoxicity in diabetic retinopathy, Müller cells also contribute to the progression of the disease by decreasing their potassium buffering capacity. In diabetic patients, the expression of potassium channels is altered in Müller cells due to the accumulation of AGEs, thus decreasing the conductance and increasing the resistance to signal transduction. The alterations in potassium homeostasis lead to neuronal dysfunction and subsequent glutamate excitotoxicity. Activation of CD40, a member of TNF receptor superfamily, induces ATP release in Müller cells and promotes the expression of ATP receptor P2X_7_ in endothelial cells. These concerted actions lead to the activation of programmed cell death in endothelial cells. The deleterious effects mediated by the activation of CD40 are necessarily triggered by Müller cells, as the activation of CD40 receptor directly in endothelial cells does not impact cell viability (Portillo et al., 2016). Besides ATP, activation of CD40 receptor in Müller cells induces the expression of iNOS, TNF, and IL-1β in microglia/macrophages, promotes retinal leukostasis, and increases protein nitration (Portillo et al., 2014, 2017). Nevertheless, in an *in vitro* model, Müller cells do not induce leukocyte deposition in endothelial cells, indicating that the involvement of Müller cells to BRB breakdown in diabetes is not due to leukocyte adhesion but due to alterations of tight junction protein expression and organization (Leal et al., 2008). Müller cells also increase VEGF expression and contribute to vessel permeability through oxidative stress and the impairment of tight junction proteins, such as occludin, claudin-5, and ZO-1 (Wang et al., 2010) (**Figure 3**).

### Microglia

Microglia are the immune cells responsible for maintaining homeostasis in the brain and retina (Du et al., 2017). Under basal conditions, microglia constantly surveil the retinal parenchyma, secreting trophic factors and phagocytizing cell debris and unneeded or dysfunctional synapses (Langmann, 2007; Madeira et al., 2015). Upon alterations in retinal function, microglial cells, in an attempt to restore homeostasis, alter their phenotype. In these circumstances, microglial cells become amoeboid, have high migratory capacity and increased phagocytic efficiency, and release pro-inflammatory factors (Langmann, 2007; Karlstetter et al., 2010; Madeira et al., 2015; Goncalves et al., 2016). The initial low-grade inflammatory profile of microglia is protective for retinal tissue by limiting the damage caused by dysfunctional neurons and endothelial and glial cells. Nevertheless, in chronic retinal diseases the sustained reactivity of microglia becomes deleterious, initiating a cascade of inflammatory events that culminate in neurodegeneration. Several reports have demonstrated the role of microglia in the loss of retinal ganglion cells and endothelial dysfunction with subsequent BRB breakdown in diabetic retinopathy (Krady et al., 2005; Gaucher et al., 2007; Zeng et al., 2008; Grigsby et al., 2014; Goncalves et al., 2016; Sorrentino et al., 2016) (**Figure 3**). Upon exposure to high glucose levels, microglia become reactive and secret pro-inflammatory mediators namely, TNF (Costa et al., 2012) and IL-1β (Krady et al., 2005; Baptista et al., 2017). These cytokines induce the dysfunction of the BRB by enhancing caspase-3 activity in endothelial cells. In the human diabetic retina, microglia become reactive and strategically distributed along vessels with altered integrity (Zeng et al., 2008; Zhang H. et al., 2009). Interestingly, in proliferative diabetic retinopathy microglia are found in the vicinity of intraretinal hemorrhages, microaneurysms, and cotton-wool spots (Zeng et al., 2008). In addition, microglia directly respond to AGEs, initiating a signaling cascade that summons leukocyte invasion to the neural retina (Grigsby et al., 2014). Evidence shows that microglia become readily activated in response to various stimuli. Activated microglia produce many cytotoxic factors, and Nox-mediated release of superoxide is a common feature in many degenerative diseases (Gao et al., 2002; Qin et al., 2002; Zhang et al., 2005; Zeng et al., 2014). Moreover, there are several reports supporting that microglia-derived ROS may be a crucial and prevailing factor of microglia activation (Gao et al., 2002; Qin et al., 2002; Block, 2008). Nox2-generated ROS in microglia is a key mediator of hypoxia-induced neovascularization (Chan et al., 2013; Zeng et al., 2014). Furthermore, a specific Nox1/4 inhibitor, GKT137831, was able to decrease ischemia-induced ROS production and a pro-inflammatory phenotype in microglia (Deliyanti and Wilkinson-Berka, 2015). Whether Nox could be a valuable therapeutic target in the management of neovascularization and inflammation in diabetic retinopathy has not yet properly addressed.

## Conclusion

Diabetic retinopathy is a multifactorial disease with a complex pathophysiology, in which excessive production of ROS and oxidative stress play a crucial role in the onset and progression of this disease. Crosstalk between oxidative stress-related and inflammatory pathways is likely to be important in inducing BRB breakdown and pathological neovascularization. A better understanding of endothelial dysfunction and the elucidation of the complex crosstalk between retinal endothelial cells and neuroglial cells and leukocytes, via both cell-to-cell contact and secretion of cytokines may facilitate the development of novel therapies with significant clinical benefit in comparison with existing therapies, namely for neovascularization reduction, but also for the initial stages of the disease. Simultaneous interventions addressing multiple metabolic and signaling pathways may be beneficial to attenuate oxidative stress and inflammation and ameliorate the retinal injury.

## Author Contributions

AS, RB, IA, and RF wrote the first draft of the manuscript. AA revised the manuscript. All authors revised and approved the final version of the manuscript.

## Conflict of Interest Statement

The authors declare that the research was conducted in the absence of any commercial or financial relationships that could be construed as a potential conflict of interest. The handling Editor declared a shared affiliation, though no other collaboration, with the authors.

## Abbreviations

AGEs: advanced glycation end products
BRB: blood–retinal barrier
CD18: integrin ligand integrin beta-2
CD40: cluster differentiation 40 receptor
ICAM-1: intercellular adhesion molecule-1
IRMA: intraretinal microvascular abnormalities
IL: interleukin
NF-κB: nuclear factor-κB
NOS: nitric oxide synthase
Nox: nicotinamide adenine dinucleotide phosphate (NADPH) oxidase
OCTA: optical coherence tomography angiography
PKC: protein kinase C
RAGE: receptor for AGEs
ROS: reactive oxygen species
SIRT1: sirtuin 1
STAT: signal transducers and activators of transcription proteins
TNF: tumor necrosis factor
VEGF: vascular endothelial growth factor
